# Advances in microRNAs as Emerging Biomarkers for Colorectal Cancer Early Detection and Diagnosis

**DOI:** 10.3390/ijms252011060

**Published:** 2024-10-15

**Authors:** Maša Ždralević, Andrijana Radović, Janja Raonić, Natasa Popovic, Aleksandra Klisic, Ljiljana Vučković

**Affiliations:** 1 Institute for Advanced Studies, University of Montenegro, Cetinjska 2, 81000 Podgorica, Montenegro; 2 Faculty of Medicine, University of Montenegro, Kruševac bb, 81000 Podgorica, Montenegronpopovic@ucg.ac.me (N.P.); aleksandranklisic@gmail.com (A.K.); ljvuckovic@gmail.com (L.V.); 3 Center for Pathology, Clinical Center of Montenegro, Ljubljanska bb, 81000 Podgorica, Montenegro; janja.histology@gmail.com; 4 Center for Laboratory Diagnostics, Primary Health Care Center, 81000 Podgorica, Montenegro

**Keywords:** microRNA, polyp, adenoma, colorectal cancer, diagnosis, treatment

## Abstract

Colorectal cancer (CRC) remains the second most common cause of cancer-related mortality worldwide, necessitating advancements in early detection and innovative treatment strategies. MicroRNAs (miRNAs), small non-coding RNAs involved in gene regulation, have emerged as crucial players in the pathogenesis of CRC. This review synthesizes the latest findings on miRNA deregulated in precancerous lesions and in CRC. By examining the deregulation patterns of miRNAs across different stages of CRC development, this review highlights their potential as diagnostic tools. We specifically analyse the roles and diagnostic relevance of four miRNAs—miR-15b, miR-21, miR-31, and miR-146a—that consistently exhibit altered expression in CRC. The current knowledge of their role in key oncogenic pathways, drug resistance, and clinical relevance is discussed. Despite challenges posed by the heterogeneity of the research findings on miRNA deregulation and their role in CRC, integrating miRNA diagnostics into current screening methods holds promise for enhancing personalized medicine approaches. This review emphasizes the transformative potential of miRNAs in CRC diagnosis, paving the way for improved patient outcomes and novel therapeutic paradigms.

## 1. Introduction

Colorectal cancer (CRC) remains a critical global health challenge, ranking as the second leading cause of cancer-related mortality and the third most frequently diagnosed cancer worldwide, with nearly two million new cases reported in 2022 [1]. European regions have the highest incidence rate of CRC, correlated with their socio-economic development and associated negative lifestyle factors and diet changes, such as decreased physical activity and the increased intake of animal-based foods [1]. Together with obesity, heavy alcohol consumption, and cigarette smoking, these factors are independently associated with an increased CRC risk [2]. Particularly worrisome is an increase in early-onset CRC incidence, in patients younger than 50 years in many countries [1], underscoring the importance of advancing early detection, accurate prognosis, and effective treatment strategies.

The majority of patients with CRC, about 75%, have sporadic disease, and the remaining 20–30% are thought to have a familial basis—a positive family history, common exposure, and/or risk factors [3]. Pathogenic variants in genes responsible for CRC are estimated to account for only 5–6% of patients, and these include familial adenomatous polyposis (FAP), hereditary nonpolyposis colorectal cancer (HNPCC), or Lynch syndrome, hamartomatous polyposis syndrome, and some other very rare syndromes [4].

The primary treatment modality for early-stage CRC is surgical resection, and pathohistological analysis of the resected tissue remains the strongest predictive factor of patients’ outcomes despite many important advances made in molecular subclassification and the development of molecular markers [5]. Among patients with metastatic CRC, the prognosis is related to the location and extent of the distant metastatic disease, and genomic profiling for *KRAS*, *NRAS*, *BRAF*, microsatellite instability, and mismatch repair deficiency is recognized in national guidelines as essential to guiding an appropriate therapeutic regimen [6,7]. Approximately 15–30% of patients present with metastatic disease, and 20–50% of patients with localized disease will develop metastases, most commonly in the liver, lung, peritoneum, and distant lymph nodes [8]. The survival rate of metastatic CRC patients is less than 20%; however, CRC progression from benign adenomas to malignant adenocarcinomas is a rather slow process, which opens a critical possibility for early detection. In clinical practice, the early and accurate detection of CRC is crucial for improving patient outcomes and survival rates. Therefore, identifying novel molecular biomarkers that could serve as diagnostic and prognostic tools, and possibly become therapeutic targets in the future, is a highly effective approach to reducing the burden of CRC.

MicroRNAs (miRNAs), small endogenous non-coding RNA molecules, have emerged as one of the most promising candidate biomarkers which could overcome the limitations of the existing screening methods and significantly improve the early detection of CRC. miRNAs act as post-transcriptional regulators of gene expression, primarily by binding to the 3′ UTR of target mRNA, leading either to the degradation of mRNA transcripts or the inhibition of mRNA translation [9]. Altered gene expression is one of the core molecular features of cancer and, knowing that miRNA represents one of the most abundant gene-regulatory molecules, it comes as no surprise that the dysregulation of miRNA expression is closely related to cancer initiation, progression, and metastasis [10]. In particular, miRNAs are critical for the regulation of the cellular stress response, and affect all hallmarks of cancer cells, with a profound impact on cell proliferation, differentiation, apoptosis, angiogenesis, pro-inflammatory signalling, metabolic reprogramming, invasion, and metastasis [11,12,13]. A single miRNA can bind up to a hundred different transcripts and, in turn, one mRNA can be regulated by various miRNAs, which can function either as a tumour suppressor or an oncogene [14]. miRNAs themselves are also regulated via chromosomal alterations at miRNA loci, epigenetic dysregulation, and alterations in miRNA-processing genes and proteins [15].

The presence of miRNAs in the circulation originates from cell death-related processes such as necrosis, apoptosis, trauma, and tumours, or from cell secretion [16], in which they act as messengers that enable long-distance and paracrine cellular communication [17]. Their broad distribution, as well as their stability and resistance to repeated freeze–thaw cycles, pH and temperature fluctuations, and ribonucleases, thanks to their binding to argonaute proteins, or their encapsulation in extracellular vesicles [18,19], makes miRNAs promising diagnostic and prognostic biomarkers in a variety of diseases [20]. Additionally, miRNAs in circulation can be identified by microRNA-specific quantitative polymerase chain reaction (PCR), thus enabling significantly greater sensitivity in their detection in comparison to protein biomarkers [21].

MiRNAs found in CRC have been extensively studied, mainly in blood and stool samples [22]. Due to their relatively high reproducibility and stability in stool, faecal miRNAs are assumed to be reliable biomarkers for CRC screening [23]. A variety of studies have examined faecal miRNA panels, faecal single miRNAs, or combinations of faecal miRNAs with faecal haemoglobin for CRC detection, showing a better diagnostic capability if faecal miRNAs and faecal haemoglobin levels are combined, as compared with faecal miRNAs or faecal haemoglobin alone [23]. Since miRNAs have a small molecular size (approximately 20–25 nucleotides), these biomarkers have also been recently examined in urine samples of CRC patients [24].

The aim of this comprehensive review is to explore the multifaceted role of miRNAs in CRC, focusing on their potential as biomarkers for diagnosis. We discuss the mechanisms by which miRNAs influence CRC pathogenesis, including their regulation of key signalling pathways such as the Wnt/β-catenin, PI3K/AKT, and MAPK pathways. We have also performed a thorough literature search of all relevant studies in which miRNAs were used for the diagnosis of precancerous lesions and CRC, highlighting recent advancements in the identification and validation of specific miRNAs as diagnostic markers. The PubMed, Embase, Web of Science, and Science Direct databases were searched from the year 2010 to the year 2024, using the following keywords: colorectal adenoma, colorectal polyp, colorectal cancer, microRNA, miRNA, diagnostic, diagnosis, detection, biomarker, blood, plasma, serum, and stool. A comprehensive analysis of selected candidate miRNAs was performed. Relevant articles were screened based on the title and abstract. For the original articles published from 2010 onwards that matched the relevant keywords, the full text was further examined. To be included, studies needed to evaluate miRNA expression in both CRC patients and control groups. Studies reporting single miRNAs, miRNA panels, and/or both were eligible. Additionally, studies had to report at least on sensitivity and specificity, or AUC, to be included. Studies published in languages other than English and those with insufficient data were excluded.

Through this in-depth analysis, we aim to provide a detailed overview of the current landscape of miRNA research in CRC, highlighting the transformative potential of these molecules in addressing the unmet clinical needs of CRC patients.

## 2. Current CRC Diagnostic Techniques

Current screening techniques for CRC include the guaiac-based faecal occult blood test (gFOBT), faecal immunochemical test (FIT), and DNA test, which detect occult blood; endoscopic examinations, including colonoscopy and flexible sigmoidoscopy, and computed tomographic (CT) colonography [25,26].

A summary of the already established screening methods is given in Table 1.

The American Cancer Society recommendations regard individuals of 45 years of age and older, whereas the United States Preventive Task Force recommendations regard individuals from 45 to 75 years of age.

The FIT exhibits higher sensitivity (76% vs. 39%) and specificity (96% vs. 94%) for CRC screening than the gFOBT. The FIT is more specific for the lower gastrointestinal tract (GIT), whereas the gFOBT shows the ability to detect bleeding from any part of the GIT [27]. Importantly, unlike the gFOBT, which can also detect substances other than blood, leading to false-positive results, the FIT only detects human blood, thus being less interfered with by medications and dietary factors [27].

A multi-target stool DNA test (Cologuard) is a non-invasive tool like other stool tests but is also not a cost-effective test [31]. It shows higher sensitivity (92% vs. 76%), but lower specificity than the FIT (87% vs. 96%) (see Table 1).

Colonoscopy is regarded as the gold standard method for CRC screening but has a series of limitations that hamper its adoption in population-wide screening [31,32]. Its sensitivity and specificity are 95% and nearly 100%, respectively [29]. However, colonoscopy is an invasive procedure, and it is not applicable to the entire population that is at risk [31]. Unlike stool tests for CRC screening, colonoscopy is inconvenient for the patient since it demands cleansing of the whole bowel, it is time-consuming, and the entire colon cannot be visualized by all examinations [33].

Flexible sigmoidoscopy needs less time for its related examination than colonoscopy and the bowel preparation is faster and easier. It is less expensive, and the rates of complications are lower than during colonoscopy. However, despite its high specificity (98–100%) for detecting distal colorectal neoplasms, its sensitivity is low (35–70%) for the entire colon since it is limited to identifying proximal colon cancer [33].

CT colonography is a radiological procedure that is applied when colonoscopy is limited and is followed by rare major complications [33]. CT colonography shows high sensitivity (90%) and specificity (88%) in CRC screening [34], but high patient radiation exposure and an inability to identify flat and small lesions are the main drawbacks of this method [33]. The pre-operative evaluation of commonly used serum tumour markers, such as CEA, CA19-9, CA-125, and AFP, is very important for the treatment planning of patients with CRC, because their levels have been associated with the prognosis of CRC patients [35]. However, none of them is recommended for CRC screening and diagnosis, and they are therefore out of the scope of this article.

Therefore, there is an utmost need for a non-invasive, cost-effective CRC screening test to enhance screening accuracy. The use of microRNAs (miRNAs) could be a promising CRC screening biomarker.

## 3. miRNAs in Diagnosis of CRC

### 3.1. Pathogenesis of CRC and miRNA

The process of CRC tumorigenesis occurs through multiple steps. The sequence of genetic changes that transform healthy colorectal epithelial cells first into adenoma, and subsequently into invasive carcinoma, was initially described by Fearon and Vogelstein in the 1990s [36]. These genetic changes are responsible for silencing tumour suppressor genes and activating oncogenes. Fearon and Vogelstein hypothesized that the accumulation of a minimum of four or five such genetic changes is necessary for the complete malignant transformation from normal to neoplastic epithelium. Interestingly, they noted that the number of genetic alterations increases proportionally with the malignant potential of the tumour, with a small percentage of early adenomas having only one, while most CRCs usually have from four to five of the described genetic alterations. Consequently, altered cell signalling through several key pathways in the pathogenesis of CRC has been described to date, such as APC/Wnt/β-catenin, p53, COX, TGF-β/SMAD, NF-κB, Notch, VEGF, and JAK/STAT3 (reviewed in [37,38]). It is important to note that, in addition to genetic alterations, the silencing of tumour suppressors and activation of oncogenes can be caused by epigenetic changes through changes in the expression and the mutation of miRNAs. In fact, altered miRNA expression was shown to affect all the above-mentioned key signalling pathways [39].

The key pathway recognized in the alteration of the normal epithelium to adenoma is mutation or epigenetic changes in the APC/Wnt/β-catenin pathway. The *APC* gene is a tumour suppressor that is frequently mutated in familial cases of CRC. In contrast, in sporadic cases of CRC, the loss of a certain part of chromosome 5, or the allelic loss of 5q, the region that normally contains the *APC* gene, is observed. The most recognized miRNAs involved in the regulation of this signalling pathway, which are therefore important players in the pathogenesis of CRC, are miR-135a, miR-135b, and miR-21 [40,41]. In addition, the *KRAS* gene mutation status is one of the key differences between early stage and late-stage adenomas that are more likely to progress to invasive carcinoma. A recent study identified 13 miRNAs whose altered expression is uniquely associated with *KRAS* mutation in patients with CRC when compared to those with the *KRAS* wild type [42]. This indicates that some of these miRNAs could be used as potential biomarkers for CRC.

The allelic loss of 17p, which contains the tumour suppressor gene *TP53*, is a hallmark feature of CRC cells but is almost never present in adenomas. Although point mutations and allelic losses of this gene are nonspecific features of CRC, as they are present in many other malignancies, their importance is reflected in the fact that they are more frequently present in tumours with high malignant potential. The miRNAs described to be involved in the regulation of p53 activity are miR-125b, miR-34a, miR-504, miR-122, miR-29 [43], miR-15a, and miR-16 [44]. Since from four to five genetic/epigenetic changes are necessary for malignant transformation, and some of them are nonspecific and occur in different malignancies, it is expected that a panel of from four to five miRNAs rather than a single miRNA will hold the most diagnostic and therapeutic potential in the future.

#### Molecular Classification of CRC

The classical adenoma–carcinoma sequence is primarily associated with the chromosomal instability (CIN) subtype of CRC, where key mutations in *APC*, *KRAS*, and *TP53* drive the progression from normal epithelium to adenoma to carcinoma. However, the microsatellite instability (MSI) and CpG island methylator phenotype (CIMP) subtypes represent alternative pathways that can also follow the adenoma–carcinoma sequence, albeit through different mechanisms involving microsatellite instability and epigenetic modifications [5]. MSI tumours arise due to defects in the DNA mismatch repair (MMR) system, leading to the accumulation of replication errors, particularly in microsatellite regions [45]. CIMP is characterized by the widespread hypermethylation of CpG islands in the promoter regions of tumour suppressor genes, leading to gene silencing [46]. This epigenetic alteration can occur early in adenoma formation. CIMP is often seen in conjunction with MSI, particularly in tumours with *BRAF* mutations. This overlap suggests that CIMP-positive adenomas can progress to carcinoma through pathways that involve both epigenetic modifications and MSI.

The consensus molecular subtypes (CMSs) provide a broader framework that incorporates these traditional and alternative pathways, highlighting the heterogeneity of CRC pathogenesis. The CMS classification, established through an integrative analysis of large-scale genomic, transcriptomic, and epigenomic data, provides a comprehensive framework for understanding the molecular diversity of CRC [47]. This classification system, endorsed by the CRC Subtyping Consortium, identifies four main subtypes of CRC, each characterized by specific biological and clinical attributes. Understanding these relationships is crucial for developing targeted therapeutic strategies and for the prevention and early detection of CRC.

### 3.2. miRNAs in the Detection of Precancerous Lesions

It is well-known that most cases of CRC arise from precursor lesions—polyps [48], which are defined as growths or protrusions into the lumen above the adjacent colonic mucosa. Conventional adenomas and serrated polyps are two main histologic types of neoplastic polyps that serve as direct precursors to most CRC [49]. The Paris classification is the most widely validated and accepted system used to describe colorectal polyp morphology in vivo and helps to categorize colorectal polyps and stratify the risk of CRC [50]. Lesions are generally classified into two main categories: polypoid (type 0-I) and non-polypoid (type 0-II). Polypoid lesions can be further divided into pedunculated (type 0-Ip) and sessile (type 0-Is) types. Non-polypoid lesions (type 0-II) are subdivided into superficially elevated (0-IIa), flat (0-IIb), or depressed (0-IIc) forms. Excavated lesions fall under type 0-III. The risk of CRC, including submucosal invasion, has been found to increase in direct proportion to the polyp size and the presence of depression [51].

The accumulation of genetic and epigenetic changes in precursor lesions over a 5–20-year period leads to the development of carcinoma [52]. In addition to the most prevalent genetic alteration—*APC* mutation leading to chromosomal instability in the classic adenoma–carcinoma sequence—two more-significant but less-prevalent pathways have been described, microsatellite instability and DNA methylation in CpG islands. The latter two fall into the category of serrated pathway [53].

Along with the above-mentioned, a growing number of studies in the past 15 years have investigated the role of miRNAs in the pathogenesis of CRC and their use as biomarkers in various biological samples. In a large number of studies, miRNA expression in CRC and healthy controls was examined, but, in contrast to this, the data on the expression of miRNAs in adenomas and their ability to trigger different pathways and expression profiles which could help to stratify the malignant potential of adenomatous lesions are scarce. In Table 2, we have summarized the findings of the studies on deregulated miRNAs in precancerous colorectal lesions.

In one of the earliest studies on this topic, Tsikitis et al., using FFPE tissue samples from different types of polyps, including hyperplastic polyps (HPs), tubular adenomas (TAs), sessile serrated adenomas (SSAs), traditional serrated adenomas (TSAs), and high grade and tubulovillous adenomas (TVHGs), found that the expression of miR-320a increased and miR-145 and miR-192 expression decreased with a higher histologic grade and proposed these three miRNAs as early biomarkers for patient risk stratification [62].

In a different study by the same group, using a larger panel of miRNAs, the authors separated serrated and non-serrated lesions. In hyperplastic/normal mucosa, relative to advanced adenomatous polyps, the most highly expressed miRNAs were miR-145, -143, -107, -194, and -26a, while miR-663, -1268, -320b, -1275, and -320b were down-regulated. In addition, the authors have shown miR-124, -143, and -30a to have a high accuracy in separating high- from low-risk polyps irrespective of the presence of serrated histology; miR-145 and -619 to be discriminatory between low- and high-risk polyps without serrated histology; and miR-335, -222 and -214 between non-serrated and serrated histology [69].

In FFPE tissue specimens, Kanth et al. identified several other miRNAs (miR-31-5p, -135b-5p, -549a, -3614-5p, -222-5p, -144-3p, -584-5p, -451a, -4488, -151a-5p, and -205-5p) to be good predictors of serrated neoplasia [74]. Ito et al. found higher expression levels of miR-31 in SSAs (including those with dysplasia) and TSAs relative to in HPs. Moreover, miR-31 expression was shown to be associated with CIMP-high status in serrated lesions with *BRAF* mutation [61]. The miR-125b levels progressively increased from normal mucosa, across low-/high-grade adenomas, to carcinomas, suggesting its association with colorectal neoplasia initiation and progression [66].

Aslam et al., also by analysing miRNAs isolated from FFPE samples, found that the loss of APC expression is associated with higher expression levels of miR-135b [66]. The up-regulation of miR135b, along with miR-21, miR-29a, and miR-92a, was also found in adenoma tissue in the study of Uratani et al. [70]. On the other hand, only the latter three miRNAs showed higher expression levels when examined in serum and correlated with the polyp size and number. The authors therefore proposed these three miRNAs as non-invasive diagnostic biomarkers for identifying high-risk adenomatous colorectal lesions [70].

Tadano et al., using FFPE tissue samples, showed a progressively decreasing expression of the miR-320 family (except miR-320d) from normal epithelia, across adenoma, to submucosal invasive carcinoma, and concluded that the miR-320 family plays an important role in colorectal tumour growth by targeting CDK6 and should be considered to be a biomarker for its early detection [68].

In their large population-based study of CRC, Slattery et al. found that miRNAs are highly deregulated in colorectal tissue and that the pattern of deregulation varied as the process progressed from normal to adenoma to carcinoma, as well in relation to the polyp type [67]. miRNAs in adenomatous polyps were more likely to be up-regulated relative to normal colonic mucosa, while miRNAs in SSPs and HPs were more likely to be down-regulated relative to normal colonic mucosa [71].

Wang et al. found miR-10a, -141, -146a, 151-3p, -194, and -3607-3p to be decreased in FFPE tissue samples of advanced adenomas, including recurrent ones, compared to normal colorectal tissue. Moreover, miR-194 was shown to be an independent predictor for adenoma recurrence in patients with advanced colorectal adenoma after endoscopic removal [63].

In the attempt to resolve the diagnostic dilemma between adenomas with epithelial misplacement and adenomas with early carcinoma, given the different clinical approaches to these patients, Žlajpah et al. analysed the expression of several extracellular matrix-related genes and proteins, and their regulatory miRNAs using RT-qPCR and immunohistochemistry in FFPE tissue samples. Their results showed that miR-146a, miR-29a, miR-29b, miR-29c, miR-200b, miR-200c, and let-7a were up-regulated in adenoma, AEM, and AEC. A significant difference between adenoma and adenoma with epithelial misplacement was found for miR-29c [76]. Moreover, they observed a negative correlation between miR-200c and decorin (DCN) expression as well as miR-146a and secreted phosphoprotein 1 (SPP1) expression, and a positive correlation between let-7a and erythropoietin-producing hepatoma receptor A4 (EPHA4) expression [76].

In frozen tissue samples from porcine models carrying a germline *APC* mutation, using next-generation sequencing, Stachowiak et al. detected several miRNAs (ssc-let-7e, ssc-miR-98, ssc-miR-126-3p, ssc-miR-146a-5p, ssc-miR-146b, ssc-miR-183, and ssc-miR-196a) associated with early-stage colorectal neoplasia, concluding that the deregulation of these miRNAs could determine progression in colorectal polyps and could be a potential target for early therapy [72].

It has been observed that alterations in the miRNA expression in tissue and plasma samples can show a similar trend. In the study of Nagy et al., three miRNAs (miR-31, -4506, and -452) were differentially expressed in adenomas when compared with adjacent mucosa, and a similar result was found in their plasma samples [77].

Examining small RNAs as biomarkers, Roberts et al. found decreased levels of miR-335-5p in the plasma of patients with adenoma [73]. Their results also suggest that miR-335-5p, along with other identified small RNAs, could have utility in adenoma detection in patients below the age of 50 [73], which could be valuable given the fact that the CRC incidence is rapidly increasing in the younger adult population [78].

Plasma samples were used in several different studies. The concentrations in plasma of miR-24, miR-320a, miR-423-5p [64], miR-601, and miR-760 [55] were all decreased in patients with adenoma and CRC, and miR18a was found to be up-regulated in colorectal neoplasia [57].

Kanaan et al. proposed a panel of eight miRNAs (miR-532-3p, miR-331, miR-195, miR-17, miR-142-3p, miR-15b, miR-532, and miR-652) to detect colorectal adenomas [56]. In another study, no significant correlation between the expression levels of several miRNA transcripts (miR-10a, -29a, -92a, -100, -125b, -196a, -17-3p, -31, -184, -187, -200b, and -203) and cases of adenoma was found [60].

A specific 6-miRNA signature (miR-15b-5p, miR-18a-5p, miR-29a-3p, miR-335-5p, miR-19a-3p, and miR-19b-3p) was found to be discriminatory between carcinoma, advanced adenoma, and healthy people, and could be detected in plasma [79] as well as in serum [75]. The combination of these six miRNAs with testing of the faecal haemoglobin concentration was therefore proposed as a strategy to improve the diagnostic accuracy of current screening methods [79].

In plasma and serum samples, miR-21 and miR-92a were found to be significantly up-regulated in patients with adenomas and CRC [54,58,59]. These two miRNAs have significant diagnostic value for advanced neoplasia.

Besides from tissue, plasma, and serum samples, miRNA profiling was also conducted in stool samples. Birkeland et al. have shown that the left-over FIT buffer used in CRC screening can be used for miRNA detection [80]. In the study of Wu et al., the authors proposed stool-based miR-135b to be used as a non-invasive biomarker for the detection of CRC and advanced adenoma [65].

Aside from the above-mentioned numerous nuclear miRs, Wallace et al. hypothesized that mitochondrial miRs (mitomiRs) could affect mitochondrial functional pathways, leading to the transition from colorectal adenoma to carcinoma, and found the expression of mitomiRs-24, -181, -210, and 378 to be progressively increased with the histologic grade [81].

### 3.3. miRNAs in the Detection of CRC

Recent advances in high-throughput sequencing and microarray technologies have facilitated the identification of numerous miRNAs with altered expression in CRC. In our review, a total of 87 papers were analysed, revealing a total of 143 deregulated microRNAs associated with CRC (Table 3). Among these, 88 microRNAs showed increased expression, while 42 showed decreased expression in CRC patients with respect to healthy controls. Additionally, 13 microRNAs demonstrated both increased and decreased expression in different studies. Notably, miR-21, miR-92a, miR-20a, miR-29a, miR-221, miR-23a, miR-223, and miR-150 were the most frequently studied, with 19, 15, 7, 5, 5, 5, 4, and 3 mentions in different studies, respectively (Table 3). miR-21 and miR-92a emerged as the most consistently studied and validated miRNAs, showing significant promise as diagnostic markers. Some studies concentrated on specific individual miRNAs, whereas others have utilized miRNA panels in an effort to increase the sensitivity and specificity for detecting CRC. A total of 24 panels with 3 or more miRNAs was found, comprising 76 different miRNAs. Importantly, all miRNAs reported in this review were validated by qRT-PCR.

Serum was the most commonly utilized biospecimen, featuring in 34 (39.1%) of the reviewed studies. Serum is a convenient and cost-effective method, but it can lack specificity for CRC since certain microRNAs can show aberrant expression in other types of cancer as well. Other types of samples that were analysed include plasma, stool, saliva, whole blood, exosomes, and urine, providing a comprehensive overview of potential sources for miRNA detection in CRC screening (Table 3). Stool samples, while potentially offering greater specificity for CRC and gastrointestinal-related miRNAs, may be less favoured due to patient reluctance to collect them. Urine collection is straightforward, but the lower concentrations of miRNAs found in urine might limit its diagnostic effectiveness [157].

The sample size varied significantly across studies, ranging from as few as 13 CRC patients and 5 healthy controls (HCs) [150], to as many as 200 CRC patients and 400 HCs [92]. Twenty-one studies included tissue samples in addition to other sample types, like serum or plasma, to validate miRNA biomarkers and understand their expression in cancerous tissues (see Table 3). These samples helped confirm the correlation between circulating miRNAs and those in tumour tissues, enhancing the reliability of the findings.

In the majority of the reviewed studies, strict criteria were used for patient selection: patients with CRC underwent preoperative colonoscopy, and adenocarcinoma was confirmed by histopathology. None of these patients had received radiotherapy or chemotherapy before blood sampling. Individuals with a history of familial adenomatous polyposis, hereditary non-polyposis CRC, or previous malignant tumours were excluded. The healthy subjects were asymptomatic individuals recruited through colonoscopy screening. This approach ensured that participants without CRC or other significant gastrointestinal conditions were included as controls in the studies.

miR-21 and miR-92a were not only the most consistently studied and validated miRNAs, but they also demonstrated the best values for sensitivity, specificity, and AUC among all analysed studies. In that regard, it is also important to mention the remarkable diagnostic accuracy found for miR-139-3p, which demonstrated 96.6% sensitivity, 97.8% specificity, and an AUC of 0.994 [112]. In the study by Roman-Canal et al., 10 analysed miRNAs also demonstrated good diagnostic performances; however, the limited sample size of this study may affect the statistical power and generalizability of the findings, making it necessary to validate these results in larger, independent cohorts before drawing definitive conclusions [156].

MiR-21 has been extensively studied in the context of CRC and is frequently highlighted in the literature. Among the 19 studies included, miR-21 demonstrated a sensitivity greater than 80% in 10 studies, a specificity greater than 80% in 9 studies, and an AUC greater than 0.800 in 12 studies (see Table 3). The highest sensitivity and specificity for miR-21 was found in a study by Sabry et al., which showed a 91.4% sensitivity and 95% specificity, with an AUC of 0.973, thus indicating serum miR-21 as a promising diagnostic marker [119]. Additionally, a study by Ghareib et al. reported strong results for miR-21, with a sensitivity and specificity of 95.8% and 91.7%, respectively, and an AUC of 0.940 [126].

MiR-92a belongs to the miR-17-92 precursor cluster, which yields five more miRNAs: miR-17, miR-18a, miR-19a, miR-20a, and miR-19b [158]. In the study by Hassan R et al., miR-92a demonstrated strong performance as a marker with a sensitivity of 94.2%, a specificity of 100%, and an AUC of 0.991 [99]. Similarly, in the research conducted by Zaki A et al., miR-92a showed a sensitivity of 98.1%, a specificity of 93.9%, and an AUC of 0.994 [100]. These studies reported the best results for the use of miR-92a as a marker compared to the other studies included in this review. However, miR-92a has been implicated in several other diseases, including liver cancer [159], breast cancer [160], and heart disease [161], showing that it is not specific to CRC. Therefore, more research is needed to fully understand its role in disease before it can be reliably used in medical practice.

miR-20a is known to be upregulated in both solid and hematopoietic cancers and has been proposed as a diagnostic serum biomarker for various cancers, including prostate [162], gastric [163], and nasopharyngeal cancers [164]. Among the seven reviewed studies, only Yang Q et al. observed the downregulation of miR-20a [118], contrary to findings in other studies. However, this inconsistency could be attributed to the study’s small sample size and population characteristics.

Studies indicate that miR-29a dysregulation plays multiple roles across various types of cancer [165]. Consistently, miR-29a can act as both a tumour suppressor and an oncogene in the development of CRC [166]. In the study by Herreros-Villanueva et al., miR-29a, along with miR-19a, miR-19b, miR-15b, miR-335, and miR-18a, demonstrated compelling results for CRC detection, achieving a sensitivity of 91%, a specificity of 90%, and an AUC of 0.950 [79]. Furthermore, three other studies investigated miR-29a and found that it acted as an oncogene, showing increased expression in CRC [54,85,123].

MiR-221 is recognized as an oncomiR, and its high expression is linked to poor patient prognosis [167]. Although the miR-221 expression levels did not show significant variation based on gender, tumour type, or stage, there was a noticeable difference related to the age of the patients noticed in the study by Cai K et al. [168]. In the studies analysed in this review, miR-221 consistently showed an elevated expression, with AUC values ranging from 0.606 to 0.882 (see Table 3).

MiR-23a is one of the top 50 deregulated miRNAs in CRC, and it promotes colon cancer cell growth, invasion, and metastasis by suppressing the expression of metastasis suppressor genes [57,82]. Additionally, increased miR-23a expression has been linked to advanced tumour stages, greater invasion depth, and lymph node metastasis, suggesting that miR-23a could serve as a potential biomarker for CRC [169]. Serum exosomal miR-23a demonstrated strong diagnostic potential for CRC, with a high sensitivity (92%) and perfect specificity (100%). It outperformed other miRNAs from the same study and traditional CRC biomarkers (CEA and CA19-9) in these aspects. Additionally, this study showed that miR-23a is effective in detecting early-stage CRC, suggesting its utility for early diagnosis [149].

The heterogeneity observed in the expression patterns of miRNAs like miR-150 and miR-223 across different studies underscores the complexity of using miRNAs as universal biomarkers. During the analysis of the collected studies, it was observed that miR-150 can be either up-regulated [149,152] or down-regulated [145] in patients with CRC. The increased expression of miR-150 has been noted not only in CRC but also in other types of cancers, such as acute myeloid leukaemia [170] and cervical cancer [171]. Similarly, miR-223 has shown variable expression patterns in CRC studies. Three studies reported the increased expression of miR-223, all showing an AUC greater than 0.700 in serum exosomes, stool, and serum samples [91,108,149]. However, Zhu Y et al. showed opposite results in faecal samples [137]. Differences in the sample type and size, the stages of patients, and natural variations in faeces might have contributed to these differences.

The exploration of miRNA panels, as opposed to individual miRNAs, offers another promising avenue for improving the sensitivity and specificity of CRC diagnostics. Panels combining multiple miRNAs have shown superior performance, with several achieving area under the curve (AUC) values greater than 0.900, indicating strong diagnostic potential. Out of 24 panels with 3 or more miRNAs, 21 demonstrated an AUC greater than 0.800. The highest-performing panel was that by Tan Y et al., which included miR-144-3p, miR-425-5p, and miR-1260b, and showed a sensitivity of 93.8%, a specificity of 91.3%, and an AUC of 0.954 [97]. Other top-performing panels include those by Radwan et al., which achieved a sensitivity of 91%, a specificity of 93%, and an AUC of 0.954 and included miR-92a, miR-211, and miR-25 [98], and by Guo S et al., with a sensitivity of 91.6%, a specificity of 91.7%, and an AUC of 0.960, including miR-1246, miR-1229-3p, miR-202-3p, miR-21-3p, and miR-532-3p [117]. miR-92a therefore confirms its utility both when used as a single marker and as a part of a panel.

While blood remains the most commonly used sample type, some researchers explored the use of urine samples for miRNA-based CRC screening as a practical alternative. Urine offers the advantage of easier collection compared to stool or blood, and the miRNAs it contains are stable under standard clinical storage conditions, thus avoiding the need for needle sticks and potentially improving patient comfort [172]. Iwasaki et al. identified higher expression levels of miR-566 and miR-129-1-3p in urine samples of CRC patients, as compared to those from healthy individuals [24]. Moreover, miR-566 and miR-129-1-3p expression levels were also higher in both tissue and sera samples of these patients, assuming that the CRC tissues’ overexpression of these miRNAs leads to their secretion into the circulation and excretion into the urine [24]. The authors presumed that urinary miR-566 and miR-129-1-3p could surpass the faecal immunochemical test (FIT) regarding CRC early detection. However, these findings need to be further validated.

**Figure 1:**
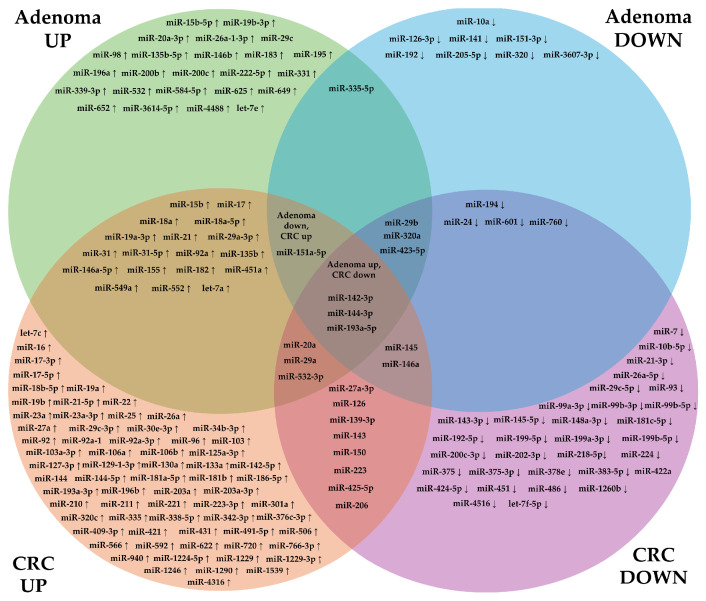
A Venn diagram of miRNAs differentially expressed in precancerous lesions and CRC. The figure highlights the up-regulation (↑) and down-regulation (↓) of miRNAs in adenomas and CRC. It distinguishes miRNAs whose expression is overlapping across the conditions. miRNAs without arrows refer to miRNAs that are both up- and downregulated.

To summarize the analysed data, in Figure 1, all precancerous lesions were grouped together in order to compare the miRNA deregulation patterns between precancerous states and CRC.

We have identified 24 miRNAs that are up-regulated and 8 miRNAs that are down-regulated only in adenomas. Only one miRNA, miR-335-5b, was found to be deregulated in both directions in adenomas. In CRC, 68 miRNAs were up- and 32 miRNAs were down-regulated, while 8 miRNAs were both up- and down-regulated. A total of 18 miRNAs was found to be up-regulated both in adenomas and in CRC, and 4 miRNAs were down-regulated in both groups. miR-20a, miR-29a, and miR-532-3p were up-regulated in adenomas, but both up- and down-regulated in CRC. On the other hand, miR-29b, miR-320a, and miR-423-5p were both up- and down-regulated in adenomas, and down-regulated in CRC. miR-145 and miR-146a were found to be down-regulated in adenomas but could be both up- or down-regulated in CRC. From the clinical point of view, maybe the most interesting are miR-151a-5p, which is down-regulated in adenomas and up-regulated in CRC, and a group of three miRNAs (miR-142-3p, miR-144-3p, and miR-193a-5p) which are up-regulated in adenomas, while their expression is down-regulated in CRC. They could potentially be good markers for precancerous lesions since their expression differs between cancer stages.

In order to give meaning and better understand the consequences of miRNAs’ deregulation in cancer, it is necessary to investigate the mechanisms behind the regulation of their expression and function, either as oncomiRs or tumour suppressors. miRNAs are known to regulate cellular processes responsible for essentially all cancer hallmarks [11], but their role is complex and context-dependent, reflecting the complexity of the cancer itself. Furthermore, miRNAs are not exclusively negative regulators of gene expression, and positive miRNA–gene correlations are found to be surprisingly common [173]. In the following section, we selected four representative miRNAs as examples of said complexity, exhibiting often contradictory findings even in the same type of cancer, warranting further investigation.

### 3.4. Comprehensive Analysis of Selected miRNAs as Promising Biomarkers for CRC

#### 3.4.1. miR-15b

Hsa-miR-15b is encoded by the *MIR15B* gene, located on the cytogenetic band 3q25.33 of chromosome 3. miR-15b is involved in the pathogenesis of several cancers, and many non-malignant conditions, including Alzheimer’s and Parkinson’s disease, atherosclerosis, coronary artery disease, myocardial infarction, and diabetic nephropathy and retinopathy [174]. In cancers, including CRC, miR-15b was shown to have both oncogenic and tumour-suppressive roles [175,176]. For example, miR-15b exerted an oncogenic role in breast cancer, promoting its proliferation, migration, and invasion by directly targeting heparanase-2 [177]. An oncogenic role of miR-15b was shown in bladder [178], cervical [179], ovarian [180], and gastric cancers [181]. On the other hand, miR-15b was shown to have a tumour-suppressive role in thyroid cancer [182], hepatocellular carcinoma [183], neuroblastoma [184], osteosarcoma [185], and prostate cancer [186]. miR-15b exerts its effects in these cancers through various mechanisms, such as the regulation of proliferation, apoptosis, epithelial–mesenchymal transition (EMT), and drug resistance, mediated by the NF-κB, STAT3, AKT/mTORC1, CDC42/PAK1, and β-catenin signalling pathways [174].

Emerging evidence indicates that miR-15b has an important role in the pathogenesis, progression, and anti-tumour therapy response of CRC. The relative expression of miR-15b in colorectal cancer cells was shown to be significantly lower than in normal cells, and spectrin beta, nonerythrocytic 2 (SPTBN2) was identified as a direct target of miR-15b [187]. Since high SPTBN2 levels were correlated with a poor prognosis in CRC patients, SPTBN2 negative regulation by miR-15b demonstrates its tumour-suppressive role. In addition, the tumour-suppressive role of miR-15b was demonstrated to occur via the inhibition of PD-L1 expression at the protein level, the inhibition of tumorigenesis, and increased anti-PD-1 sensitivity in murine models of CRC [188]. It is known that drug resistance is one of the critical factors related to treatment failure, and miR-15b was shown to be an important mediator in 5-fluorouracil (5-FU) resistance in CRC. Namely, mir-15b overexpression improved the sensitivity of colorectal cancer cells to 5-FU by enhancing cell apoptosis by targeting NF-kB1 and one of its kinase complexes, IKK-α [176]. Furthermore, miR-15b overexpression suppressed tumorigenic properties of tumour-initiating cells and restored sensitivity to adjuvant chemotherapy and neoadjuvant radiotherapy in CRC patients by targeting doublecortin-like kinase 1 (*DCLK1*), a putative gastrointestinal stem cell marker [189].

On the other hand, the inhibition of miR-15b transcription by sirtuin 1 (SIRT1) deacetylase decreased metastasis in CRC animal models [190]. The authors identified peroxisomal acyl-CoA oxidase 1 (ACOX1) as a direct target of miR-15b, implying the important role of altered lipid metabolism in CRC metastasis. The oncogenic role of miR-15b in CRC was also shown by Gasparello et al. [175], who demonstrated that the downregulation of miR-15b in the HT-29 CRC cell line correlated with growth inhibition and the activation of apoptosis.

Similarly, reports on miR-15b expression levels in CRC patient samples were contradictory [174], underscoring the importance of further investigation of its role in CRC, since it has been identified to have a high diagnostic accuracy for CRC [153,191]. In our analysis, miR-15b expression was found to be up-regulated both in adenomas and in CRC groups.

#### 3.4.2. miR-21

miR-21 is one of the most studied miRNAs involved in the pathophysiology of CRC. The MIR21 gene is located within the vacuole membrane protein 1 (VMP1) locus on chromosome 17. VMP1 was shown to be negatively correlated with the CRC prognosis, and its loss of expression led to an aggressive form of CRC [192]. Although widely regarded as oncogenic, there are some reports on its tumour-suppressive role in CRC as well [193]. miR-21-knock out reduced tumour development in vivo by negatively regulating the expression of *Spry1*, *Pten*, and *Pdcd4*, but miR-21-null mice did not have any other phenotypic anomalies [194]. miR-21 exerts its oncogenic role by inhibiting the expression of several well-known tumour suppressor genes, such as phosphatase and tensin homolog (*PTEN*) [195], and programmed cell death 4 (*PDCD4*) [196], which in turn alter the expression of their downstream targets, leading to the increased invasion, intravasation, and metastasis of CRC cells. Several other studies provided insights into miR-21-mediated oncogenic mechanisms in CRC: miR-21 induced pyroptosis in CRC cells by targeting transforming growth factor beta-induced (*TGFBI*) [193]; it suppressed Krev interaction protein 1 (KRIT1) and activated the β-catenin signalling pathway in endothelial cells, thereby promoting angiogenesis and vascular permeability [197]; miR-21 overexpression promoted proliferation and invasion and inhibited apoptosis in CRC cells by targeting the Ras homolog gene family, member b (RhoB) [198]; it promoted tumour growth partially by down-regulating sec23a expression [199]; and miR-21 knock-down was associated with the increased expression of Sprouty2, a tumour suppressor gene, which reduced the proliferation rate of CRC cells [200]. Importantly, miR-21 expression was found to be associated with an increase in CRC stroma, compared to the normal tissue, and ectopic stromal miR-21 expression was related to an increased invasiveness, highlighting the importance of the deregulation of stromal miRNAs for CRC progression [201]. miR-21 was shown to be implicated in the regulation of glycolysis, apoptosis, autophagy, epithelial-mesenchymal transition, drug resistance, and resistance to radiotherapy in different types of cancer [202], which warrants further exploration of its role in CRC.

A recent meta-analysis of the diagnostic potential of miR-21 in CRC showed it had a pooled 79% sensitivity and 92% specificity [203], making it a good candidate for further exploration of both its role as a part of a miRNA panel for CRC diagnosis in an independent cohort, and its functional role in CRC initiation. The expression of miR-21 in this study was found to be up-regulated both in adenomas and in CRC, which could challenge its implementation in the clinic.

#### 3.4.3. miR-31

miR-31 has a dual role in many human cancers, but it acts as an oncogenic miRNA in CRC [204]. miR-31 stimulates CRC proliferation and tumorigenesis through the inhibition of RASA1 translation, and the consequent activation of the Ras signalling pathway [205], and is shown to facilitate CRC migration and invasion, together with miR-21 [206]. The transcription of miR-31 is induced by IL-1β, via the p38/JNK pathways, and miR-31 binds and directly targets E-selectin, thereby modulating the metastatic process [207,208]. The effect of miR-31 is anti-metastatic, since miR-31 inhibition increases the adhesion and transendothelial migration of colon cancer cells [207]. miR-31 expression was found to be up-regulated in *BRAF*-mutated (*V600E*) CRC, compared to the wild-type *BRAF* carriers, indicating it to be an independent unfavourable prognostic factor, and to correlate with SSA/P and TSA, confirming its oncogenic role in the serrated pathway [209]. Enhancer of zeste homolog 2 (EZH2), a methyltransferase that plays a critical role in the regulation of CRC invasion and metastasis, was shown to suppress miR-31 expression in CRC and to correlate with the evolution of the serrated pathway [210]. Further confirming its role in SSA evolution was the finding that a high miR-31 expression correlated with CpG island methylator phenotype (CIMP)-high status in serrated lesions with a *BRAF* mutation, thus placing miR-31 as an important molecule supporting the colorectal continuum concept [61]. miR-31 inhibition in vitro had an antitumour effect, thus placing miR-31 among potential therapeutic targets against CRC [209]. The expression of miR-31 was also shown to be increased in CRC patients harbouring *KRAS* mutations, compared to patients without these mutations [211]. Numerous miR-31 targets testify to the importance of its role in CRC tumorigenesis, such as factor inhibiting HIF-1α (FIH-1) [212], cyclin-dependent kinase inhibitor 2B (CDKN2B) [213], T lymphoma and metastasis gene 1 (TIAM1) [206], SATB homeobox 2 (SATB2) [214], paired box 6 (PAX6) [215], tensin 1 (TNS1) [216], Rho-related BTB domain containing 1 (RhoBTB1) [217], cell death inducing p53 target 1(CDIP1) [218], NUMB endocytic adaptor protein [219], STX12, eIF4EBP1, and eIF4EBP2 [220], and, in these studies, the role of miR-31 was proven to be both oncogenic and tumour-suppressive. The function of miR-31 therefore depends highly on its interactions with other factors in the TME and is context-dependent, which is supported by the existence of the broad spectrum of its molecular targets [221]. In addition, miR-31 was found to have an important role in TME; namely, high miR-31 expression in cancer-associated fibroblasts inhibited autophagy, suppressed migration, and increased the radiosensitivity of co-cultured colorectal cancer cells [222]. Conditional miR-31 knock-out was demonstrated to result in more severe colitis-associated CRC with respect to the wild-type, thus promoting tumour development [223].

The clinical significance of altered miR-31 levels is reflected in its potential to serve as a diagnostic [224,225], a prognostic [226,227,228,229,230], and a biomarker of lymph node metastasis [231], as well as to regulate drug [232,233,234] and radiation sensitivity [235]. miR-31 was found to be upregulated in colorectal adenomas with respect to controls [77], and, in this study, it proved to be up-regulated both in adenomas and in CRC. In addition, high miR-31 expression correlated with an advanced tumour stage and poor differentiation [229,236], as well as a deeper invasion of CRC tumours [237]. In the metastatic setting, miR-31 was proven to be a valuable potential prognostic biomarker for anti-EGFR therapy, since high miR-31 expression was associated with a shorter PFS in these patients, carrying all wild-type genes [238,239]. In three other studies, miR-31 expression was significantly associated with PFS in *KRAS* wild-type mCRC patients treated with anti-EGFR therapy [240,241,242], but not when the tumour was right-sided [243], and in these patients, miR-31 was also associated with the time to progression [244].

#### 3.4.4. miR-146a

miR-146a was shown to both promote and inhibit CRC tumorigenesis [245,246,247]. It is strongly implicated in inflammatory signalling and the immune response; namely, miR-146a was identified as a major negative regulator of CRC tumorigenesis by modulating IL-17 responses and thereby limiting tumorigenic inflammation [248]. In human CRC cells, miR-146a was shown to be poorly expressed; consequently, its ectopic expression inhibited the proliferation, migration, and invasion of CRC cells, suggesting its tumour-suppressive role [245]. On the other hand, miR-146a was found to be up-regulated in CRC tissues and to have an oncogenic role [246]. By directly targeting carboxypeptidase M (CPM), miR-146a was proposed to promote cell migration and invasion by regulating the c-Src, a non-receptor tyrosine kinase, and focal adhesion kinase (FAK) expression [246]. Consistently, miR-146a was found to regulate the division of spheroid-derived CRC stem cells by targeting Numb, a tumour suppressor and segregation determinant, activating the Wnt signalling pathway and promoting tumorigenicity [249]. Furthermore, exosomal miR-146a and miR-155 were found to promote C-X-C motif chemokine receptor 7 (CXCR7)-mediated CRC metastasis by increasing the levels of the inflammatory cytokines interleukin-6, tumour necrosis factor-α, transforming growth factor-β, and CXCL12 [250]. The activation of cancer-associated fibroblasts (CAFs) with miR-146a and miR-155-5p was shown to promote the invasion and formation of lung metastasis in vivo using tumour xenograft models [250]. Furthermore, miR-146a overexpression in HT-29 CRC cells was shown to induce resistance to chemotherapeutic drugs, 5-FU, and irinotecan [251]. The miR-146a polymorphism rs2910164 was shown to be associated with the susceptibility to and prognosis of CRC [252]. The expression levels of miR-146a are altered in serum [122] and the tissue samples of CRC patients [253], and patients with high miR-146a levels were shown to have better overall survival [254]. Serum miR-146a was shown to have a significant diagnostic ability in CRC as a member of a three-miRNA panel, together with miR-30e-3p, and miR-148a-3p [122]. In our pilot study, we found significantly increased miR-146a expression both in the tumour tissue and plasma of the same patients with CRC [255], and in this review, miR-146a was found to be down-regulated in adenomas and both up- and down-regulated in CRC, which warrants the further investigation of miR-146a expression in larger patient cohorts and further elucidation of its role in CRC development and diagnosis.

## 4. Discussion

Despite the existing knowledge of CRC pathogenesis and risk factors, as well as established screening programs, this malignancy still represents a significant public health problem, being the most common cancer in Western countries and the second leading cause of cancer-related deaths [1]. The detection of patients in the early stages, including the detection of precancerous lesions, which is a key point in survival, is still insufficient.

Along with clarified genetic mechanisms of CRC carcinogenesis, there has been an increasing number of studies over the past 15 years dedicated to the role of epigenetic events, particularly focusing on the role of miRNAs in this process. Publications addressing the role of miRNAs in the initiation of the neoplastic cascade and transition from precancerous lesion to adenoma/polyp to CRC, their role as non-invasive biomarkers in the detection of early neoplastic lesions, and the risk stratification of these patients relative to miRNAs’ expression are relatively scarce and highly heterogeneous in many ways. Studies on this topic encompass various biological samples, ranging from frozen and FFPE tissue samples to plasma, serum, urine, saliva, and stool specimens. It is apparent that miRNAs exhibit a similar pattern of deregulation across different biological samples, and there is an increasing trend towards the application of non-invasive techniques for their detection [75,77]. In previously mentioned studies on precancerous lesions, miR-21, miR-29, and miR-92 show up-regulation in plasma, serum, and tissue samples; miR-31 and miR-18 are overexpressed both in stool and tissue samples; and miR-135b is up-regulated in plasma, stool, and tissues.

Moreover, highly varied panels of miRNAs were included in this investigation, where some researchers relied on previously published studies of miRNA expression in CRC pathogenesis [60,66,70,75], while others proposed panels obtained via high throughput studies [56,62,65,67,68,69,72,73,77].

Additionally, we have noticed different nomenclature and sample groupings in different studies. For example, in one study [68], the Paris and Japanese classifications of colorectal lesions was used, while others mostly relied on the WHO classification of digestive system tumours. In the latter group of studies, some researchers unified all colorectal polyps into the category of adenoma (vs. normal tissue/carcinoma) [67,68,73], while others sub-classified adenomas based on their histological grade into early and advanced [56,60,65,66,70,75,77,84], the latter ones being larger than 1 cm and having villous or tubulovillous histology, or high-grade dysplasia [256]. In addition, considering the multiple pathways of carcinogenesis described in the CRC pathogenesis, including the serrated pathway, miRNA expression in relation to the presence of a serrated morphology in colorectal polyps was analysed in only a few studies [61,62,69,71,74]. Through these studies, it has been observed that certain miRNAs can discriminate between serrated and non-serrated aetiologies [62,69,71], and that miRNAs are more likely to be up-regulated in adenomatous polyps and down-regulated in serrated lesions in relation to normal colonic mucosa. Interestingly, some miRNAs, such as miR135b, may be involved in both the serrated pathway and the classic adenoma–carcinoma sequence in the same manner [65,66,70,74].

Not including serrated lesions in this study group is reasonable to a certain extent, considering that some serrated lesions such as traditional serrated adenomas are very rare, representing only about 1% of all colorectal polyps, and the fact that the malignant potential of some serrated lesions was previously underestimated. It is known that SSLs with dysplasia and TSAs are the most common precursors of CRC. On the other hand, HPs are most commonly small, asymptomatic lesions, and have minimal malignant potential. However, it was observed that HPs could progress to SSLs or TSAs for a period of 7.5 years and, in this context, predispose to CRC [257].

It is important to note that most of the aforementioned studies were conducted before the publication of the 5th edition of the WHO Classification of Tumours of the Digestive System, in which serrated lesions’ classification in particular, underwent many changes [258]. According to the current WHO classification, using strict criteria, serrated lesions/polyps are classified into four categories: hyperplastic polyps (HPs), sessile serrated lesions (SSLs), traditional serrated adenoma (TSAs), and unclassified serrated adenomas [258]. In the earlier classifications, serrated lesions were not well defined and often grouped together with HPs or misclassified due to significant morphological overlap.

Given the aforementioned points, the heterogeneity of biological samples, the different panels of miRNAs investigated, and the different nomenclatures and groupings of precancerous lesions as well as the changes made in their classification, a more precise systematization of miRNAs in precancerous lesions is yet to be established. However, it is important to stress that miRNAs show great promise in detecting precancerous lesions more effectively than current tests, such as the FIT and gFOBT, which have rather low sensitivity for adenoma detection (17% and 23%, respectively) [25]. In contrast, several miRNAs have shown superior sensitivity and specificity in detecting precancerous lesions (see Table 2). For instance, miR-21 and miR-320a have demonstrated high diagnostic accuracy, with studies reporting sensitivities of 91.9% and 92.79% and specificities of 81.1% and 73.08%, respectively. From a clinical standpoint, the integration of miRNA-based diagnostics into existing CRC screening programs holds the potential to enhance early detection while overcoming some of the limitations of current methods, such as sensitivity and scalability. Combining miRNA testing with established protocols, such as using miRNAs as a follow-up test after a positive FIT result or as part of a multi-modal approach with stool DNA testing, could increase their diagnostic accuracy and reduce the need for invasive procedures.

The findings of this review underscore the potential of miRNAs as valuable biomarkers for CRC diagnosis and treatment. With 143 deregulated miRNAs identified across 87 studies, the extensive deregulation observed reflects the significant role that miRNAs play in CRC pathogenesis. However, the variability in miRNA expression profiles across different studies highlights the inherent complexity of miRNA regulation in cancer and the need for critical evaluation of these biomarkers before clinical implementation.

MiR-21 and miR-92a stand out as consistently deregulated and well-studied miRNAs, often associated with a high sensitivity and specificity for CRC detection. However, the use of these miRNAs as standalone diagnostic markers is challenged by their involvement in other diseases, suggesting that they may not be exclusively specific to CRC. This raises concerns about their specificity and potential false-positive rates in clinical settings. Therefore, while miR-21 and miR-92a show promise, their clinical utility may be limited unless used in combination with other miRNAs to form a diagnostic panel that can offer a more specific and sensitive tool.

The exploration of miRNA panels, which combine multiple miRNAs, offers a promising approach to overcoming the limitations of individual miRNA markers. Panels have shown higher diagnostic accuracy and could address the issue of variability seen in single-miRNA studies. Nonetheless, the development of these panels must be approached with caution. The choice of miRNAs included in the panels should be based on rigorous validation studies, considering not only their expression in CRC but also their potential roles in other conditions that could confound diagnostic results.

The selection of biospecimens for miRNA analysis is another critical aspect that requires careful consideration. While serum is a convenient and commonly used sample type, its use may lack the specificity required for CRC due to miRNA alterations in other cancers and diseases. Alternative biospecimens such as stool, urine, and exosomes may offer an increased specificity but come with their own challenges, such as patient compliance and lower miRNA concentrations. Certainly, further research is needed to optimize sample collection, processing, and storage methods to ensure the reliability and reproducibility of miRNA-based diagnostics.

The comprehensive analysis of miRNA expression patterns in precancerous lesions and CRC reveals distinct miRNA deregulation profiles, suggesting the dynamic roles of these molecules in the progression from adenomas to malignancy. Notably, the identification of 24 miRNAs that are up-regulated and 8 miRNAs that are down-regulated specifically in adenomas underscores the possibility that these miRNAs play a role in the early stages of colorectal tumorigenesis. The presence of a single miRNA, miR-335-5b, that exhibits bidirectional deregulation in adenomas may indicate its complex regulatory function in early neoplastic changes. In CRC, the marked increase in deregulated miRNAs, with 68 being up-regulated and 32 being down-regulated, illustrates the extensive reprogramming of miRNA expression as the disease progresses to malignancy. The observation of 18 miRNAs being up-regulated and 4 being down-regulated in both adenomas and CRC suggests a continuum in miRNA expression changes from precancerous to cancerous states, implicating these miRNAs in the neoplastic transformation process. Interestingly, the divergent expression patterns of specific miRNAs between adenomas and CRC, such as that of miR-151a-5p, which is down-regulated in adenomas and up-regulated in CRC, and miR-142-3p, miR-144-3p, and miR-193a-5p, which are up-regulated in adenomas but down-regulated in CRC, highlight their potential as biomarkers that can differentiate between precancerous and cancerous stages. These miRNAs, given their contrasting expression profiles, could be pivotal in distinguishing early lesions from more advanced CRC, thus aiding in early detection and intervention strategies.

The heterogeneity observed in miRNA expression patterns across different studies presents a major challenge. Factors such as the sample size, the patient demographics, and methodological differences contribute to this variability, indicating a need for standardized protocols in miRNA research. Large-scale, multicentre studies with well-defined patient populations and standardized methodologies are essential to validate the clinical utility of miRNAs as biomarkers for CRC.

Understanding the functional roles of miRNAs in CRC is crucial for elucidating their potential as diagnostic and therapeutic targets. While miRNAs are known to regulate key signalling pathways involved in cancer, their exact roles in CRC remain to be fully elucidated. The four miRNAs chosen to be represented in this review, miR-15b, miR-21, miR-31, and miR-146a, are intricately involved in CRC pathogenesis, with each miRNA demonstrating both unique and overlapping roles in tumour regulation. The dual nature of miRNAs, acting as both oncogenes and tumour suppressors, complicates their therapeutic targeting. As an example, miR-15b promotes apoptosis and enhances drug sensitivity, but also promotes metastasis through altering lipid metabolism. miR-21 is predominantly an oncomiR, promoting tumorigenesis by inhibiting tumour suppressors such as PTEN and PDCD4, and facilitating invasion, metastasis, and chemoresistance. Its up-regulation in both adenomas and CRC indicates its involvement from the early stages of tumorigenesis, but at the same time, this complicates its clinical interpretation, despite its high sensitivity and specificity as a diagnostic marker. Additionally, the involvement of miR-21 in stromal interactions and regulation of the TME suggests that targeting miR-21 could disrupt critical oncogenic pathways in CRC. miR-31 also has a well-documented role as an oncogenic miRNA in CRC due to its ability to activate the Ras signalling pathway, promote migration and invasion, and regulate key factors involved in metastasis. Its expression correlates with *BRAF* and *KRAS* mutations, poor differentiation, and advanced tumour stages, marking it as a significant prognostic marker. The predictive value of miR-31 for an anti-EGFR therapy response in metastatic CRC patients highlights its relevance to personalized medicine. miR-146a plays a significant role in modulating inflammation and immune responses in CRC, which are critical aspects of CRC pathogenesis. Its dual role is indicative of its context-dependent effects. The ability of miR-146a to regulate IL-17 responses and limit tumorigenic inflammation positions it as a potential target for immunomodulatory therapies. Conversely, its promotion of metastasis through the activation of CAFs and induction of chemoresistance raises concerns about its oncogenic potential. The contradictory findings regarding miR-146a expression in CRC and its varied roles in different studies highlight the need for a deeper understanding of its regulatory mechanisms and interactions within the tumour microenvironment.

In summary, while miRNAs hold great promise as non-invasive biomarkers for CRC, significant challenges remain. Rigorous validation, the careful selection of biospecimens, and a deeper understanding of miRNA biology are necessary to fully realize their potential in clinical practice. Continued research and collaboration are essential to overcome these challenges and translate the promise of miRNAs into effective tools for CRC diagnosis, but also CRC prognosis and treatment.

## 5. Conclusions and Future Directions

The dynamic interplay between miRNAs and their targets has opened new opportunities for the development of diagnostic biomarkers. While significant progress has been made in identifying and validating miRNAs as potential CRC biomarkers, many challenges need to be addressed before these can be fully integrated into clinical practice. The variability in miRNA expression patterns, the need for more comprehensive validation studies, and the optimization of sample types are key areas that require further investigation. Nonetheless, the use of miRNAs, particularly in combination panels, holds considerable promise for enhancing the early detection and diagnosis of CRC, ultimately contributing to improved patient outcomes.

Future efforts should focus on the incorporation of miRNA profiling into standard diagnostic procedures. However, a thorough understanding of miRNAs’ biology and function in CRC must precede these efforts, given their multifaceted roles and complex interactions with TME.

## Figures and Tables

**Table 1:** Current screening tests for CRC. ACS—American Cancer Society; USPTF—United States Preventive Task Force; FOBT—faecal occult blood test; CT—computed tomographic.

Screening Method	Biological Sample	Mechanism of Action	Sensitivity and Specificity	Reference	ACS Recommendations	USPTF Recommendations
**Guaiac FOBT**	Stool	Detects blood	39% 94%	[27]	Annually	Annually
**Immunochemical** **FOBT**	Stool	Detects blood	76% 96%	[27]	Annually (if guaiac is not done)	Annually (if guaiac is not done)
**Stool DNA** **(Cologuard)**	Stool	Detects abnormal DNA and blood	92% 87%	[28]	Every 3 years	Every 1–3 years
**Colonoscopy**	Tumour tissue from anywhere in the entire colon	Direct visualization and biopsy/removal, requires bowel preparation	95% 100%	[29]	Every 10 years	Every 10 years
**Flexible** **sigmoidoscopy**	Tumour tissue only from the rectum and sigmoid	Direct visualization and biopsy/removal, requires bowel preparation	35–70% 98–100%	[30]	Every 5 years	Every 5 years
**CT colonography**	No sample is taken	Visualization of the colon, requires bowel preparation	90% 88%	[28]	N/A	Every 5 years

**Table 2:** The list of studies involving miRNAs as diagnostic markers for precancerous colorectal lesions. AP—adenomatous polyp, HP—hyperplastic polyp, TSA—traditional serrated adenoma, SSA—sessile serrated adenoma, AEM—adenoma with epithelial displacement, AEC—adenoma with early carcinoma, AC—advanced carcinoma, CRC—colorectal cancer; HC—healthy controls, HG—high grade, LG—low grade, IEN—intraepithelial neoplasia, LST—laterally spreading tumour.

Article	Year	Biospecimen	Sample Size	miRNAs Deregulated	Sensitivity %	Specificity %	AUC (95% CI)
[54]	2010	Plasma	AP: 37 CRC: 120 HC: 59	miR-29a ↑ miR-92a ↑ miR-29a + miR-92a AP vs. CRC	69 84 73	89.1 71.2 79.7	0.844 (0.786–0.903) 0.838 (775–0.900) 0.773 (0.669–0.877)
[55]	2012	Plasma	AP: 100 (plasma 19 tissue) CRC: 43 (plasma) HC: 68 (plasma)	miR-601 ↓ miR-760 ↓	69.2 80	72.4 72.4	0.747 (.666–0.828) 0.788 (0.714–0.862)
		Tissue		no statistically significant results	/	/	/
[56]	2013	Plasma	Screening phase: AP: 9 CRC: 20 HC: 12 Validation phase: AP: 16 CRC: 45 HC: 26	miR-15b ↑ miR-142-3p ↑ miR-155 ↑ miR-21 ↑ miR-532 ↑ miR-331 ↑ miR-652 ↑ miR-195 ↑ miR-532-3p ↑ miR-29a ↑ miR-29c ↑ miR-423-5p ↑ miR-17 ↑ miR-193a-5p ↑ miR-339-3p ↑ AP vs. HC miR-532-3p + miR-331 + miR-195 + miR-17 + miR-142-3p + miR-15b + miR-532 + miR-652 ↑	88	64	0.868 (0.76–0.98)
[57]	2013	Plasma	AP: 60 CRC: 63 HC: 73	miR18a ↑ in AA	/	/	0.64 (0.52–0.75)
[58]	2013	Serum Tissue	AP: 43 (serum) CRC: 198 (serum), 174 (tissue) HC: 65 (serum), 174 (tissue)	miR-21 ↑ miR-21 ↑	91.9 /	81.1 /	0.919 (0.867–0.958) /
[59]	2013	Serum	AP: 50 CRC: 200 HC: 80	miR-21 ↑ miR-92a ↑ miR-21 + miR-92 ↑	65 65.5 68	85 82.5 91.2	0.802 (0.752–0.852) 0.786 (0.728–0.845) 0.847 (0.803–0.891)
[60]	2014	Plasma	AP: 73 (non-advanced); 49 (advanced) CRC: 6 HC: 48	miR-10a, miiR-31, miR-100b, miR-184, miR-187-5p, miR-196-a, miR-203, miR-29, miR-92a, miR- 17-3p, miR-125b, miR-200b panel examined. No correlation with AP found.	/	/	/
[61]	2014	FFPE	AP: 222 HP: 132 TSA: 101 without dysplasia; 16 with HG dysplasia SSA: 122 without dysplasia; 10 with dysplasia CRC: 870	miR-31 ↑ in SSA, SSA with HG dysplasia, TSA	/	/	3.04 (1.88–4.97)
[62]	2014	FFPE	AP: 66 (non-advanced); 40 (advanced) HP: 23 TSA: 11 SSA: 13	miR-320a ↑ miR-145 ↓ miR-192 ↓ (with higher histologic grade)	/	/	/
[63]	2014	FFPE	AP: 127 non-recurrent; 100 recurrent HC: 37	miR-10a ↓ miR-141 ↓ miR-146a ↓ miR-151-3p ↓ miR-194 ↓ miR-3607-3p ↓	43 69 62 79 71 68	83.5 60.6 60.6 45.7 78 71.7	0.655 (0.589–0.717) 0.643 (0.577–0.705) 0.631 (0.565–0.694) 0.648 (0.582–0.710) 0.755 (0.694–0.810) 0.696 (0.632–0.755)
[64]	2015	Plasma	AP: 59 CRC: 111 HC: 130	miR-24↓ miR-320a↓ miR-423-5p↓	78.38 92.79 91.89	83.85 73.08 70.77	0.839 (0.787–0.892) 0.886 (0.845–0.926) 0.833 (0.780–0.887)
[65]	2015	Stool Frozen tissue	AP: 110 non-advanced; 59 advanced CRC: 104 HC: 109	miR-31 ↑ miR -135b ↑ miR-20a-3p ↑ miR-182 ↑ miR-649 ↑ miR-26a-1-3p ↑ miR-625 ↑ miR-18a ↑ miR-20a ↑ miR-552 ↑ in advanced AP mir-135b ↑ in CRC and AP	/	/	0.79 (of mir-135b for CRC) 0.71 (for adenomas)
[66]	2015	FFPE	HP: 11 AP: 34 non-advanced; 10 advanced CRC: 13 HC: 11	Progressive miR-135b ↑ with lesion grade	/	/	/
[67]	2016	FFPE	AP: 290 CRC: 1893 HC: 1893	Around 600 miRNAs differentially expressed among groups	/	/	/
[68]	2016	FFPE	18 LST (3 CRC and 15 CRC with adenoma) 3 protruded CRC with adenoma	Progressive miR320 ↓ family with grade	/	/	/
[69]	2016	FFPE	AP: 26 non-advanced; 40 advanced HP: 23 TSA: 11 SSA: 13	99 miRNAs differing in at least one histopathologic group	/	/	/
[70]	2016	FFPE, total serum, and exomes from serum	AP: 27 (FFPE) 26 (serum) HC: 20 (FFPE) 47 (serum) CRC: 19	AP vs. HC total serum: miR-21 ↑ miR-29a ↑ miR-92a ↑ exomal serum: miR-21 ↑	73.1 72 65.4 69.8	68.1 66 78.7 80	0.755 (0.640–0.848) 0.676 (0.556–0.781) 0.747 (0.632–0.842) 0.770 (0.654–0.861)
[71]	2017	FFPE	AP: 277 HP: 15 SSA: 14	70 miRNAs differentially expressed among groups	/	/	/
[72]	2017	Freshly frozen tissue and FFPE	LG-IEN: 24 HG-IEN: 24 HC: 12	ssc-let-7e ↑ miR-98 ↑ miR-146a-5p ↑ miR-146b ↑ miR-183 ↑ miR-196a ↑ ssc-miR-126-3p ↓ in HG-IEN	/	/	/
[73]	2018	Plasma	AP: 94 (discovery cohort) 76 (validation cohort) HC: 95 (discovery cohort) 64 (validation cohort)	miR-335-5p ↓ un-annotated small RNA ↑	/	/	Discovery cohort: 0.711 (0.638–0.784) Validation cohort: 0.755 (0.672–0.838)
[74]	2019	Plasma	AP: 14 HP: 12 SSA: 6 HC: 56	SSA: miR 31–5p ↑ miR-135b-5p ↑ miR-549a ↑ miR-3614–5p ↑ miR-222-5p ↑ miR-144–3p ↑ miR-584–5p ↑ miR-451a ↑ miR 4488 ↑ miR-151a-5p ↓ mir-205-5p ↓ AP: miR-135b-5p ↑ miR-549a ↑ miR-584–5p ↑ HP: miR -4488 ↑	/	/	/
[75]	2019	Serum	AP: 74 CRC: 59 HC: 80	Serum levels AP miR-29a-3p ↑ miR-19a-3p ↑ miR-335-5p ↑ AP vs. HC miR-15b-5p + miR-18a-5p + miR-29a-3p + miR-335-5p + miR-19a-3p + miR 19b-3p	81	63	0.80 (0.72–0.87)
[76]	2020	FFPE	AP: 10 AEM: 13 AEC: 10 AC: 11 HC:21	AP, AEM, AEC: miR-200-b ↑ miR 200c ↑ let7a ↑ miR-29a ↑ miR-29b ↑ miR-29c ↑194 miR-146-a ↑ AC: hsa-miR-146a ↓ hsa-miR-29b ↓ miR-200-b ↑ miR-200c ↑ miR-let7a ↑ miR-29a ↑ miR-29c ↑	/	/	/

The signs “↑” and “↓” denote the direction of miRNA deregulation, and refer to the “up-” and “downregulation”, respectively.

**Table 3:** The list of studies involving miRNAs as diagnostic markers for CRC. CRC—colorectal cancer; HC—healthy controls; EVs—extracellular vesicles; PLF—peritoneal lav-age fluid. Sign “+” designates a miRNA panel.

Article	Year	Biospecimen	Sample Size	miRNAs Deregulated	Sensitivity %	Specificity %	AUC (95% CI)
[82]	2010	Plasma	CRC: 90 HC: 50	miR-17-3p ↑ miR-92 ↑	64 89	70 70	0.717 (0.630–0.800) 0.885 (0.830–0.940)
[83]	2010	Plasma	CRC: 103 HC: 37	miR-221 ↑	86	41	0.606 (0.490–0.720)
[54]	2010	Plasma	CRC: 100 HC: 59	miR-29a ↑ miR-92a ↑ miR-29a + miR-92a ↑ ^1^	69 84 83	89.1 71.2 84.7	0.844 (0.786–0.903) 0.838 (0.775–0.900) 0.883 (0.830–0.937)
[84]	2012	Plasma	Training cohort CRC: 30 HC: 30	miR-21 ↑	90	90	0.820
			Validation cohort CRC: 20 HC: 20	miR-21 ↑	90	90	0.910
[55]	2012	Plasma	CRC: 90 HC: 58	miR-601 ↓ miR-760 ↓	69.2 80	72.4 72.4	0.747 (0.666–0.828) 0.788 (0.714–0.862)
[56]	2013	Plasma	CRC: 45 HC: 26	miR-139-3p ↑ + miR-431 ↑	91	57	0.829 (0.730–0.930)
[85]	2013	Plasma	CRC: 80 HC: 144	miR-18a +miR-20a + miR-21 + miR-29a + miR-92a + miR-106b + miR-133a + miR-143 + miR-145 + miR-181b + miR-342-3p + miR-532-3p ↑	/	/	0.745 (0.708–0.846)
[57]	2013	Plasma	CRC: 42 HC: 53	miR19a + miR19b ↑ miR19a + miR19b + miR15b ↑	78.6 78.6	77.4 79.3	0.820 (0.730–0.900) 0.840 (0.760–0.920)
[86]	2014	Plasma	Training cohort CRC: 55 HC: 57	miR-7 ↓ + miR-93 ↓ + miR-409-3p ↑	91	88	0.866
			Validation cohort CRC: 22 HC: 27	miR-7 ↓ + miR-93 ↓ + miR-409-3p ↑	82	89	0.897
[87]	2014	Plasma	CRC: 94 HC: 46	miR-375 ↓ miR-206 ↑ miR-375 ↓ + miR-206 ↑	76.92 / /	64.63 / /	0.749 (0.654–0.844) 0.705 (0.612–0.799) 0.846 (0.775–0.917)
[88]	2015	Plasma	CRC: 100 HC: 79	miR-106a ↑ miR-20a ↑	74 46	44.4 73.4	0.605 (0.522–0.688) 0.590 (0.507–0.674)
[89]	2015	Plasma	CRC: 61 HC: 24	miR-142-3p ↓ miR-26a-5p ↓	/ /	/ /	0.710 (0.594–0.825) 0.670 (0.552–0.787)
[64]	2015	Plasma	CRC: 111 HC: 130	miR-24 ↓ miR-320a ↓ miR-423-5p ↓ miR-24 + miR-320a + miR-423-5p ↓	78.4 92.8 91.9 92.8	83.9 73.1 70.8 70.8	0.839 (0.787–0.892) 0.886 (0.845–0.926) 0.833 (0.780–0.887) 0.899 (0.867–0.938)
[90]	2016	Plasma	CRC: 187 HC: 47	miR-96 ↑	65.4	73.3	0.740 (0.650–0.831)
[91]	2016	Plasma	Training cohort CRC: 62 HC: 62	miR-92a ↑ miR-223 ↑	/ /	/ /	0.833 (0.763–0.904) 0.734 (0.646–0.823)
		Plasma + stool	Validation cohort CRC:153 HC:121	miR-92a ↑ miR-223 ↑ miR-92a + miR-223 ↑ miR-92a + miR-223 ↑	/ / 75.8 96.8	/ / 70.5 75	0.751 (0.693–0.808) 0.707 (0.646–0.768) / 0.907
[92]	2016	Plasma	CRC: 200 HC: 400	miR-29b ↓	61.4	72.5	0.743
[93]	2016	Plasma	CRC: 31 HC: 34	miR-21 ↑	65	85	/
[94]	2017	Plasma	CRC: 56 HC: 70	miR-506 ↑ miR-4316 ↑ miR-506 + miR-4316 ↑	60.7 83.9 76.8	76.8 60.9 75	0.747 (0.662–0.820) 0.744 (0.658–0.817) 0.751 (0.666–0.824)
[95]	2018	Plasma	CRC: 67 HC: 134	miR-21 + miR-25 + miR-18a + miR-22 ↑	67	90	0.930
[96]	2018	Plasma	Training cohort CRC: 40 HC: 40	miR-182 ↑ miR-20a ↑ miR-182 + miR-20a ↑	/ / /	/ / /	0.929 (0.875–0.983) 0.801 (0.695–0.906) 0.905 (0.841–0.968)
			Validation cohort CRC: 50 HC: 50	miR-182 ↑ miR-20a ↑ miR-182 + miR-20a ↑	78 / /	91 / /	0.891 (0.821–0.961) 0.736 (0.631–0.842) 0.831 (0.746–0.914)
[79]	2019	Plasma	CRC: 96 HC: 100	miR-19a + miR-19b + miR-15b + miR-29a + miR-335 + miR-18a ↑	91	90	0.950 (0.903–0.991)
[97]	2019	Plasma	CRC:48 HC: 47	miR-27a-3p ↓ miR-143-3p ↓ miR-144-3p ↓ miR-148a-3p ↓ miR-424-5p ↓ miR-425-5p ↓ miR-1260b ↓ miR-144-3p + miR-425-5p + miR-1260b ↓	75 72.9 93.8 79.2 79.2 83.3 81.3 93.8	85 78.7 78.7 91.5 93.6 91.5 83.3 91.3	0.881 (0.816–0.946) 0.777 (0.682–0.873 0.887 (0.815–0.959) 0.871 (0.795–0.947) 0.919 (0.863–0.975) 0.910 (0.852–0.969) 0.848 (0.766–0.929) 0.954 (0.914–0.994)
[98]	2021	Plasma	CRC: 44 HC: 40	miR-92a ↑ miR-211 ↑ miR-25 ↑ miR-92a + miR-211 + miR-25 ↑	71 71 75 91	67 90 85 93	0.766 0.794 0.812 0.954
[99]	2021	Plasma	CRC: 52 HC: 20	miR-21 ↑ miR-92a ↑ miR-21 + miR-92a ↑	90.4 94.2 96.1	100 100 100	0.977 0.991 0.981
[100]	2022	Plasma	CRC: 54 HC: 15	miR-92a ↑	98.1	93.9	0.994
[101]	2019	Plasma Exosomes from plasma	Training cohort CRC: 30 HC: 30	miR-103a-3p + miR-127-3p + miR-151a-5p + miR-17-5p + miR-181a-5p + miR-18a-5p + miR-18b-5p ↑	96.7	53.3	0.762 (0.642–0.882)
			Testing cohort CRC: 79 HC: 76	miR-103a-3p + miR-127-3p + miR-151a-5p + miR-17-5p + miR-181a-5p + miR-18a-5p + miR-18b-5p ↑	85.3	35.1	0.824 (0.758–0.889)
			Validation cohort CRC: 30 HC: 26	miR-103a-3p ↑ miR-127-3p ↑ miR-151a-5p ↑ miR-17-5p ↑ miR-181a-5p ↑ miR-18a-5p ↑ miR-18b-5p ↑ miR-103a-3p + miR-127-3p + miR-151a-5p + miR-17-5p + miR-181a-5p + miR-18a-5p + miR-18b-5p ↑	/ / / / / / / 76.9	/ / / / / / / 86.7	0.759 (0.702–0.816) 0.729 (0.669–0.788) 0.737 (0.678–0.796) 0.742 (0.684–0.800) 0.736 (0.676–0.796) 0.777 (0.722–0.832) 0.781 (0.726–0.837) 0.895 (0.813–0.977)
[102]	2012	Serum	CRC:32 HC:39	miR-21 ↑	87.5	74.4	0.850 (0.760–0.940)
[58]	2013	Serum	CRC: 186 HC: 53	miR-21 ↑	82.8	90.6	0.927 (0.886–0.956)
[59]	2013	Serum	CRC: 200 HC: 80	miR-21↑ miR-92a ↑ miR-21 + miR-92 ↑	65 65.5 68	85 82.5 91.2	0.802 (0.752–0.852) 0.786 (0.728–0.845) 0.847 (0.803–0.891)
[103]	2014	Serum	CRC: 40 HC: 40	miR-21 ↑	77	78	0.870 (0.780–0.950)
[104]	2014	Serum	CRC: 146 HC: 60	miR-155 ↑	58.2	95	0.776 (0.714–0.837)
[105]	2014	Serum	Training cohort CRC: 160 HC: 94	miR-19a-3p ↑ miR-92a-3p ↑ miR-223-3p ↑ miR-422a ↓ miR-19a-3p ↑ + miR-92a-3p ↑ + miR-223-3p ↑ + miR-422a ↓	/ / / / /	/ / / / /	0.849 0.871 0.890 0.843 0.960
			Validation cohort CRC: 117 HC: 102	miR-19a-3p ↑ + miR-92a-3p ↑ + miR-223-3p ↑ + miR-422a ↓	84.3	91.6	0.951 (0.907–0.978)
[106]	2015	Serum	CRC: 55 HC: 55	miR-194 ↓ miR-29b ↓	72 77	80 75	0.850 (0.790–0.930) 0.870 (0.800–0.960)
[107]	2015	Serum	CRC: 84 HC: 32	miR-103 ↑ miR-720 ↑	55.9 58.3	75 56.3	0.662 0.630
[108]	2016	Serum	CRC: 100 HC:24	miR-17 ↑ miR-19a ↑ miR-20a ↑ miR-223 ↑	/ / / /	/ / / /	0.813 (0.589–1.000) 0.825 (0.611–1.000) 0.788 (0.558–1.000) 0.838 (0.627–1.000)
[109]	2016	Serum	Training cohort CRC: 80 HC: 80	miR-23a-3p + miR-27a-3p + miR-142-5p + miR-376c-3p ↑	87.5	81	0.922
			Validation cohort CRC: 203 HC: 100	miR-23a-3p + miR-27a-3p + miR-142-5p + miR-376c-3p ↑ miR-23a-3p ↑ miR-27a-3p ↑ miR-142-5p ↑ miR-376c-3p ↑	88.7 / / / /	81 / / / /	0.922 0.891 0.697 0.815 0.654
[110]	2016	Serum	CRC: 211 HC: 57	miR-1290 ↑	70.1	91.2	0.830
[111]	2017	Serum	CRC: 40 HC: 40	miR-21 ↑	86.05	72.97	0.783
[112]	2017	Serum	CRC: 117 HC: 90	miR-139-3p ↓ miR-622 ↑	96.6 87.8	97.8 67.5	0.994 (0.987–1.000) /
[113]	2017	Serum	CRC: 73 HC:45	miR-206 ↓	80	82.2	0.846
[114]	2017	Serum	CRC: 64 HC:27	miR-92a ↑ miR-375 ↓ miR-760 ↓	84.4 78.1 92.2	100 100 100	0.844 (0.755–0.933) 0.781 (0.680–0.883) 0.922 (0.856–0.988)
[115]	2017	Serum	Training cohort CRC: 30 HC: 30	miR-19a-3p + miR-21-5p + miR-425-5p ↑	/	/	0.886 (0.803–0.968)
			Testing cohort CRC: 136 HC: 90	miR-19a-3p + miR-21-5p + miR-425-5p ↑	/	/	0.768 (0.706–0.831)
			Validation cohort CRC: 30 HC: 18	miR-19a-3p + miR-21-5p + miR-425-5p ↑	/	/	0.830 (0.708–0.952)
[116]	2017	Serum	CRC: 103 HC: 100	miR-196b ↑	63	87.4	0.814 (0.755–0.873)
[117]	2018	Serum	CRC: 107 HC: 120	miR-1246 ↑ miR-1229-3p ↑ miR-202-3p↓ miR-21-3p ↓ miR-532-3p ↓ miR-1246 ↑ + miR-1229-3p ↑ + miR-202-3p ↓ + miR-21-3p ↓ + miR-532-3p ↓	64.2 67.5 69.2 90.7 60.8 91.6	68.2 92.5 88.3 78.3 96.3 91.7	0.681 (0.612–0.750) 0.776 (0.713–0.839) 0.815 (0.756–0.873) 0.878 (0.831–0.924) 0.743 (0.674–0.811) 0.960 (0.937–0.983)
[118]	2018	Serum	CRC: 26 HC: 33	miR-20a ↓ miR-486 ↓	/ /	/ /	0.676 0.629
[119]	2018	Serum	CRC: 35 HC: 101	miR-210 ↑ miR-21 ↑ miR-126 ↓	88.6 91.4 88.6	90.1 95 50.5	0.934 (0.873–0.995) 0.973 (0.946–1.000) 0.665 (0.571–0.759)
[120]	2020	Serum	CRC: 148 HC: 68	miR-92a-1 ↑	81.8	95.6	0.914
[121]	2020	Serum	CRC: 110 HC: 90	miR-378e ↓	89	80	0.930 (0.897–0.962)
[122]	2020	Serum	CRC: 80 HC: 88	miR-30e-3p ↑ miR-31-5p ↑ miR-34b-3p ↑ miR-146a-5p ↑ miR-148a-3p ↓ miR-192-5p ↓ miR-30e-3p ↑ + miR-31-5p ↑ + miR-34b-3p ↑+ miR-146a-5p ↑ + miR-148a-3p ↓ + miR-192-5p ↓ miR-30e-3p ↑ + miR-146a-5p ↑ + miR-148a-3p ↓	/ / / / / / 84.6 80	/ / / / / / 86.1 78.7	0.731 (0.654–0.808) 0.669 (0.586–0.751) 0.785 (0.715–0.855) 0.739 (0.665–0.813) 0.648 (0.559–0.737) 0.652 (0.569–0.735) 0.932 (0.895–0.970) 0.883 (0.831–0.935)
[123]	2020	Serum	CRC: 73 HC:18	miR-21 ↑ miR-29a ↑ miR-92a ↑ miR-221 ↑	72.6 / / /	70.6 / / /	0.756 (0.6388–0.8728) 0.696 0.506 0.615
[124]	2020	Serum	CRC: 50 HC: 50	miR-18a ↑ miR-21 ↑ miR-92a ↑ miR-18a + miR-21 ↑	84 84 66 88	84 90 68 92	0.906 0.918 0.672 0.966
[125]	2020	Serum	CRC: 37 HC: 30	miR-1246 ↑ miR-451 ↓	100 73	80 80	0.924 0.757
[126]	2020	Serum	CRC: 48 HC: 48	miR-21 ↑	95.8	91.7	0.940
[127]	2020	Serum	CRC: 27 HC: 45	miR-21 ↑ miR-92a ↑ miR-221 ↑ miR-21 + miR-92a + miR-221 ↑	/ / / /	/ / / /	0.913 (0.848–0.978) 0.809 (0.694–0.924) 0.882 (0.804–0.960) 0.891 (0.818–0.965)
[128]	2020	Serum	CRC: 60 HC: 30	let-7c ↑ miR-21 ↑ miR-26a ↑ miR-146a ↑ let-7c + miR- 21 + miR-26a + miR-146a miR-21 + miR-26a	77.6 80.7 77.6 78 82.1 91.8	96.2 100 96.2 74.1 100 91.7	0.855 (0.770–0.941) 0.936 (0.884–0.989) 0.918 (0.857–0.979) 0.805 (0.708–0.903) 0.950 (0.898–1.002) 0.953 (0.908–0.999)
[129]	2020	Serum	CRC: 35 HC: 35	miR-21 ↑ miR-23a ↑ miR-27a ↑ miR-21 + miR-23a ↑ miR-21 + miR-27a ↑ miR-21 + miR-23a + miR-27a ↑	82.9 82.9 42.9 82.9 88.6 82.9	97.1 91.3 88.6 97.1 85.7 97.1	0.893 (0.804–0.981) 0.887 (0.802–0.973) 0.665 (0.532–0.797) 0.908 (0.822–0.989) 0.899 (0.810–0.987) 0.908 (0.824–0.993)
[130]	2020	Serum	CRC: 80 HC: 80	miR-203a-3p ↑ miR-145-5p ↓ miR-375-3p ↓ miR-200c-3p ↓ miR-203a-3p ↑ + miR-145-5p ↓ + miR-375-3p ↓ + miR-200c-3p ↓	/ / / / 81.3	/ / / / 73.3	0.712 (0.633–0.791) 0.754 (0.678–0.830) 0.715 (0.637–0.793) 0.656 (0.568–0.743) 0.893 (0.846–0.940)
[131]	2020	Serum	Training cohort CRC: 15 HC: 15	miR-592 ↑	86.6	73.4	0.880 (0.750–0.990)
			Validation cohort CRC: 134 HC: 50	miR-592 ↑	82.8	78	0.844 (0.780–0.910)
[132]	2020	Serum	CRC: 80 HC: 50	miR-4516 ↓ miR-21-5p ↑ miR-4516 ↓ + miR-21-5p ↑	94.4 90.6 92.1	89.8 86.2 87.6	0.958 0.928 0.943
[133]	2024	Serum	CRC: 46 HC: 46	miR-549a ↑ miR-552 ↑ miR-592 ↑	/ / /	/ / /	0.863 0.946 0.884
[134]	2013	Stool	CRC: 117 HC: 10	miR-106a ↑	34.2	97.2	/
[65]	2014	Stool	CRC: 104 HC: 109	miR-135b ↑	78	68	0.790
[135]	2014	Stool	CRC: 198 HC: 198	miR-221 ↑ miR-18a ↑ miR-221 + miR-18a ↑	62 61 66	74 69 75	0.730 (0.680–0.780) 0.670 (0.620–0.720) 0.750
[136]	2016	Stool	CRC: 51 HC: 26	let-7f-5p ↓	/	/	0.709 (0.591–0.827)
[137]	2016	Stool	CRC: 80 HC: 51	miR-29a ↓ miR-223 ↓ miR-224 ↓	85 60 75	61 71 63	0.777 (0.695–0.859) 0.649 (0.551–0.746) 0.744 (0.658–0.829)
[91]	2016	Stool	Training cohort CRC: 62 HC: 62	miR-223 ↑ miR-92a ↑	/ /	/ /	0.787 (0.705–0.869) 0.739 (0.651–0.828)
			Validation cohort CRC: 76 HC: 247	miR-223 ↑ miR-92a ↑ miR-223 + miR-92a ↑	77 61 71.7	65 82 79.9	0.796 (0.734–0.858) 0.748 (0.683–0.814) /
[138]	2016	Stool	CRC: 198 HC: 198	miR-20a ↑ miR-20a + miR-92a ↑ miR-20a + miR-135b ↑	55 57 79	82 84 65	0.730 (0.680–0.780) 0.770 (0.720–0.820) 0.790 (0.740–0.830)
[139]	2016	Stool	CRC: 150 HC: 98	miR-21 ↑ miR-146a ↓ miR-21 ↑ + miR-146a ↓	90.3 77.2 87	75.2 68.1 81.7	0.877 (0.810–0.972) 0.794 (0.669–0.913) 0.878 (0.779–0.965)
[111]	2017	Stool	CRC: 40 HC: 40	miR-21 ↑	86.06	81.08	0.829
[140]	2017	Stool	CRC: 29 HC: 115	miR-144-5p ↑ + miR-451a ↑	66	95	0.890 (0.820–0.950)
[141]	2019	Stool	CRC: 29 HC: 29	miR-21 ↑ miR-92a ↑ miR-144 ↑ miR-17-3p ↑ miR-92a + miR-144 ↑	79.3 89.7 78.6 67.9 96.6	48.3 51.7 66.7 70.8 37.9	0.690 (0.550–0.830) 0.760 (0.630–0.880) 0.770 (0.614–0.904) 0.710 (0.572–0.855) /
[142]	2019	Stool	CRC: 67 HC: 217	miR-421 + miR-27a-3p ↑	96	33	0.740
[143]	2019	Saliva	CRC: 51 HC: 37	miR-186-5p ↑ miR-29a-3p ↑ miR-29c-3p ↑ miR-766-3p ↑ miR-491-5p ↑ miR-186-5p + miR-29a-3p + miR-29c-3p + miR-766-3p + miR-491-5p ↑	/ / / / / 72	/ / / / / 66.7	0.655 (0.542–0.768) 0.631 (0.514–0.747) 0.659 (0.545–0.773) 0.631 (0.513–0.748) 0.632 (0.515–0.750) 0.754 (0.652–0.855)
[144]	2013	Whole blood	CRC: 70 HC: 32	miR-338-5p + miR-23a + miR-193a-3p ↑	80	84.4	0.887 (0.821–0.953)
[145]	2016	Whole blood	CRC: 71 HC: 80	miR-21 ↑ miR-221 ↑ miR-150 ↓ miR-21 ↑ + miR-221 ↑ + miR-150 ↓	71.8 71.8 57.8 80	67.5 68.8 56.3 74	0.740 0.754 0.632 0.818
[146]	2017	Exosomes from plasma	CRC: 50 HC: 50	miR-125a-3p ↑ miR-320c ↑	/ /	/ /	0.685 (0.559–0.803) 0.598 (0.471–0.726)
[147]	2018	Exosomes from plasma	Training cohort CRC: 40 HC: 40	miR-27a ↑ miR-130a ↑ miR-27a + miR-130a ↑	75 82.5 82.5	77.5 62.5 75	0.773 (0.669–0.876) 0.742 (0.633–0.851) 0.846 (0.762–0.930)
			External validation cohort CRC: 50 HC: 50	miR-27a ↑ miR-130a ↑ miR-27a + miR-130a ↑	80 70 80	77.5 80 90	0.746 (0.659–0.833) 0.697 (0.610–0.784) 0.801 (0.712–0.870)
			Validation cohort CRC: 80 HC: 40	miR-27a ↑ miR-130a ↑ miR-27a + miR-130a ↑	80 70 80	77.5 80 90	0.820 (0.742–0.899) 0.787 (0.704–0.871) 0.898 (0.844–0.953)
[148]	2020	Exosomes from plasma	CRC: 80 HC: 23	miR-139-3p ↓	/	/	0.726 (0.603–0.848)
[149]	2014	Exosomes from serum	CRC: 88 HC: 11	let-7a ↑ miR-1224-5p ↑ miR-1229 ↑ miR-1246 ↑ miR-150 ↑ miR-21 ↑ miR-223 ↑ miR-23a ↑	50 31.8 22.7 95.5 55.7 61.4 46.6 92	90.9 100 100 90.9 100 90.9 90.9 100	0.670 0.610 0.614 0.948 0.758 0.798 0.716 0.953
[150]	2019	Exosomes from serum	CRC: 13 HC: 5	miR-23a ↑ miR-301a ↑	/ /	/ /	0.890 (0.740 -1.000) 0.840 (0.650–1.000)
[151]	2019	Exosomes from serum	CRC: 165 HC: 153	miR-99b-5p ↓ miR-150-5p ↓	32.1 75.2	90.8 58.8	0.628 (0.567–0.689) 0.707 (0.649–0.764)
[152]	2020	Exosomes from serum	CRC: 45 HC: 4	miR-19a ↑ miR-20a ↑ miR150 ↑ miR-143 ↓ miR-145 ↓ let-7a ↑	/ / / / / /	/ / / / / /	0.870 0.830 0.750 0.760 0.780 0.710
[153]	2021	Exosomes from serum	Test cohort CRC: 123 HC: 150	miR-15b ↑ miR-16 ↑ miR-21 ↑ miR-31 ↑ miR-15b + miR-21 + miR-31 ↑	/ / / / 91.6	/ / / / 97.6	0.860 (0.820–0.910) 0.580 (0.510–0.650) 0.750 (0.690–0.810) 0.750 (0.680–0.820) /
			Validation cohort CRC: 81 HC: 90	miR-15b + miR-21 + miR-31 ↑	95.1	94.4	/
[154]	2021	Exosomes from serum	CRC: 51 HC: 49	miR-1539 ↑	92.2	40.8	0.673 (0.568–0.779)
[155]	2021	Exosomes from serum	CRC: 100 HC: 35	miR-126 ↑ miR-1290 ↑ miR-23a ↑ miR-940 ↑ miR-126 + miR-1290 + miR-23a + miR-940 ↑	84 85 91 90 90	88.6 88.6 74.3 77.1 88.6	0.940 (0.900–0.980) 0.920 (0.870–0.970) 0.890 (0.830–0.950) 0.880 (0.820–0.940) 0.950 (0.910–0.990)
[156]	2019	EVs from PLF	CRC: 19 HC: 22	miR-150-5p ↑ miRNA-199b-5p ↓ miR-29c-5p ↓ miR-218-5p ↓ miR-99a-3p ↓ miR-383-5p ↓ miR-199a-3p ↓ miR-193a-5p ↓ miR-10b-5p ↓ miR-181c-5p ↓	93.6 96.8 94.3 90.5 97.6 94 92 85.2 87.5 85.9	89.9 96.4 94.4 92.1 90 93.8 88.7 89.7 86.6 80.3	0.978 (0.959–0.996) 1.000 0.973 (0.954–0.991) 0.970 (0.945–0.995) 0.970 (0.950–0.990) 0.968 (0.952–0.985) 0.968 (0.942–0.994) 0.962 (0.932–0.991) 0.957 (0.930–0.983) 0.952 (0.929–0.974)
[24]	2022	Urine	CRC: 63 HC: 63	miR-129-1-3p ↑ miR-566 ↑ miR-129-1-3p + miR-566	/ / 88.9	/ / 76.2	0.856 (0.789–0.924) 0.809 (0.733–0.885) 0.868 (0.806–0.931)

^1^ When a direction of deregulation is given at the end of the panel, it refers to all miRNAs in the panel. The signs “↑” and “↓” denote the direction of miRNA deregulation, and refer to the “up-” and “downregulation”, respectively.

## Data Availability

No new data were created or analyzed in this study. Data sharing is not applicable to this article.
